# Effects of High-Protein Nutritional Guidance on Sarcopenia-Related Parameters in Individuals Aged ≥ 75 Years with Type 2 Diabetes: An Exploratory Single-Arm Pre–Post Intervention Study

**DOI:** 10.3390/nu17213459

**Published:** 2025-11-01

**Authors:** Hidechika Todoroki, Takeshi Takayanagi, Risa Morikawa, Yohei Asada, Shihomi Hidaka, Yasumasa Yoshino, Izumi Hiratsuka, Megumi Shibata, Ayumi Wada, Shiho Asai, Akemi Ito, Kosei Kamimura, Yuuka Fujiwara, Hitoshi Kuwata, Yoshiyuki Hamamoto, Yusuke Seino, Atsushi Suzuki

**Affiliations:** 1 Department of Endocrinology, Diabetes and Metabolism, Fujita Health University School of Medicine, Toyoake 470-1192, Japan; todo0808@fujita-hu.ac.jp (H.T.); morisa@fujita-hu.ac.jp (R.M.); y-asada@fujita-hu.ac.jp (Y.A.); sakai220@fujita-hu.ac.jp (S.H.); yoshi-y@fujita-hu.ac.jp (Y.Y.); idumi630@fujita-hu.ac.jp (I.H.); megumi03@fujita-hu.ac.jp (M.S.); seinoy@fujita-hu.ac.jp (Y.S.); aslapin@fujita-hu.ac.jp (A.S.); 2 Food and Nutrition Services Department, Fujita Health University Hospital, Toyoake 470-1192, Japan; harada-a@fujita-hu.ac.jp (A.W.); shiho@fujita-hu.ac.jp (S.A.); akemi305@fujita-hu.ac.jp (A.I.); 3 Nursing Department, Fujita Health University Hospital, Toyoake 470-1192, Japan; kousei@fujita-hu.ac.jp; 4 Yutaka Seino Distinguished Center for Diabetes Research, Kansai Electric Power Medical Research Institute, Kyoto 604-8436, Japan; fujiwara.yuuka@a2.kepco.co.jp (Y.F.); kuwata-kob@umin.ac.jp (H.K.); hamamoto.yoshiyuki@b4.kepco.co.jp (Y.H.)

**Keywords:** high-protein diet, glucagon, sarcopenia, older adults, type 2 diabetes mellitus, FGF21

## Abstract

**Background:** Sarcopenia and metabolic deterioration are major health concerns in adults aged ≥ 75 years with type 2 diabetes (T2DM), a population characterized by anabolic resistance, reduced dietary intake, and limited renal reserve. Optimizing protein nutrition may support muscle maintenance in this high-risk group, but clinical evidence for individualized high-protein guidance in the oldest-old population remains limited. **Objective:** We investigated whether an 18-month dietary intervention improves muscle mass and strength in adults aged ≥ 75 years with T2DM and whether serum amino acid (AA) and hormonal profiles reflect these changes. **Methods:** In this 18-month, single-arm, prospective intervention study, 44 community-dwelling adults aged ≥ 75 years with T2DM received individualized, dietitian-led nutritional guidance targeting a protein intake of approximately 1.4 g/kg ideal body weight (IBW)/day. Assessments at baseline and every 6 months included body composition, muscle strength, renal function, and fasting serum amino acid and hormonal profiles. Longitudinal changes were analyzed using paired *t*-tests and linear mixed-effects models. This trial was registered in the UMIN Clinical Trials Registry (UMIN000044687). **Results:** Skeletal muscle index and grip strength showed significant improvements at specific time points during follow-up (both *p* < 0.05), while gait speed improved at 6 months. Renal function remained clinically stable (eGFRcreat slope: +0.18 mL/min/1.73 m^2^/year; eGFRcys slope: −2.97 mL/min/1.73 m^2^/year), with no significant increase in CKD stage. Changes in glucagon correlated positively and C-peptide negatively with changes in skeletal muscle index, whereas glucagon was inversely associated with grip strength. Serum fibroblast growth factor 21 (FGF21) levels decreased over time, suggesting metabolic adaptation to the intervention. **Conclusions:** Individualized high-protein nutritional guidance for 18 months improved sarcopenia-related parameters, including skeletal muscle index and grip strength, without clinically significant deterioration of renal function in adults aged ≥ 75 years with T2DM. These findings support the feasibility and safety of protein-focused dietary counseling as a strategy to preserve muscle health in advanced age.

## 1. Introduction

The growth of the aging population has led to a substantial increase in elderly individuals with type 2 diabetes mellitus (T2DM) worldwide, a group often suffering from multiple comorbidities such as sarcopenia, osteoporosis, and cognitive impairment. In Japan, approximately 70% of patients with T2DM are aged ≥65 years, and the decline in physical and cognitive function among these individuals often results in loss of independence and reduced healthy life expectancy. In particular, adults aged ≥ 75 years exhibit accelerated declines in muscle mass, physical function, and renal capacity compared with those aged 65–74 years, making sarcopenia prevention in this population especially challenging.

Based on these characteristics, the present study focused on individuals aged ≥ 75 years, who represent the “old-old” population according to current gerontological classifications in Japan. Notably, sarcopenia has been reported to be approximately three times more prevalent in individuals with T2DM compared to those without diabetes [1]. In addition, East Asians are characterized by less obesity and insulin secretion compared to Caucasians [2], suggesting the possibility of increased susceptibility to sarcopenia. Comprehensive strategies to maintain musculoskeletal health and function in this population are urgently needed.

We focused on adults aged ≥ 75 years because the prevalence and adverse impact of sarcopenia escalate markedly beyond this age. Community-based data indicate an approximate prevalence of 22% among individuals aged 75–79 years, with sarcopenia conferring substantially higher risks of mortality and disability [3]. In parallel, chronic kidney disease (CKD) becomes significantly more common after age 75 (≈34.6% vs. 11.8% at 65–74 years) [4], making the balance between anabolic efficacy and renal safety particularly relevant in this age group. Moreover, age-related anabolic resistance—the blunted muscle protein synthesis response to protein or exercise—emerges as a key physiological constraint in advanced age [5]. Studying adults aged ≥ 75 years therefore provides a rigorous model for testing whether individualized, protein-focused counselling can preserve muscle mass while maintaining renal safety at the point of greatest clinical need.

Moreover, elderly patients with long-standing T2DM often experience progressive renal impairment, prompting dietary restrictions including reduced protein and sodium intake. In patients with moderate to severe chronic kidney disease (CKD), a protein-restricted diet (typically less than 0.8 g/kg/day) is often recommended to slow the decline in estimated glomerular filtration rate (eGFR) and reduce nitrogenous waste production [6]. Nutritional management therefore plays a critical role in preventing sarcopenia among older adults. The European Society for Clinical Nutrition and Metabolism (ESPEN) recommends a protein intake of at least 1.0 g/kg/day in older individuals to preserve muscle mass and function [7]. However, the implementation of high-protein nutritional guidance in older adults with diabetes remains debated because of concerns regarding renal load and metabolic safety.

While higher protein consumption can help preserve muscle mass and function, prior reports have also indicated potential adverse effects on kidney function at higher intake levels. Most prior trials on high-protein strategies in older adults have been short-term (≤6 months) and/or conducted in inpatient or rehabilitation settings, limiting generalizability to community-dwelling outpatients—especially those with diabetes [8,9,10,11].

To address this gap, we implemented an 18-month, individualized, dietitian-delivered program in adults aged ≥ 75 years with type 2 diabetes, thereby testing durability and renal safety in a real-world outpatient context [12].

Therefore, this study aimed to evaluate the effects of individualized high-protein nutritional guidance on both muscle-related and renal outcomes in adults aged ≥ 75 years with type 2 diabetes. Given the complex interrelation between amino acid metabolism, renal function, and muscle health, this study was designed as an exploratory, integrative analysis to generate hypotheses for future confirmatory research.

Insulin and amino acids (AAs), particularly branched-chain amino acids (BCAAs), are essential regulators of skeletal muscle protein synthesis [13,14]. In contrast, glucagon, a counter-regulatory hormone to insulin, is markedly stimulated by protein intake and plays a central role in hepatic AAs catabolism, thereby contributing to systemic AAs homeostasis [15,16,17,18]. Thus, not only nutrients but also nutrient-stimulated hormones such as glucagon regulate AAs metabolism [19]. As a result, plasma AAs concentrations are tightly regulated by dietary protein intake and the coordinated actions of insulin and glucagon. Furthermore, fibroblast growth factor 21 (FGF21), which is regulated by protein intake [20], is a hepatokine involved in energy expenditure, glucose metabolism, and the regulation of body composition [21]. Circulating and muscle-derived FGF21 is now recognized as a key regulator of muscle metabolism and mitochondrial function, acting through the mTOR–AMPK signaling axis to maintain energy balance and protein synthesis.

Elevated FGF21 reflects nutritional stress or anabolic inefficiency, such as that observed in sarcopenia, frailty, and insulin resistance, whereas its reduction may indicate improved metabolic homeostasis following nutritional intervention.

As summarized by Li et al. [22], FGF21 integrates endocrine and myogenic pathways that regulate mitochondrial stress, insulin signaling, and myogenic differentiation.

Therefore, we evaluated FGF21 as a representative metabolic stress biomarker to explore whether high-protein nutritional guidance can influence these adaptive processes.

These hormonal factors and AAs profiles may reflect an individual’s protein nutritional status as well as influence skeletal muscle mass and function. Thus, in T2DM, patients, dysregulated glucagon secretion, hepatic steatosis, and altered AAs profiles all may contribute to alterations in skeletal muscle mass and bone metabolism. The interrelationship among dietary protein intake, pancreatic/hepatic hormonal signaling, hepatic function, AAs metabolism, and musculoskeletal health in elderly patients with T2DM remains insufficiently explored.

This study evaluates muscle mass and strength and potential declines in renal and liver function under long-term dietary intervention in Japanese T2DM patients aged ≥ 75 years. We also investigate serum AAs and hormonal profiles as reflective of muscle mass and strength in these patients.

## 2. Materials and Methods

### 2.1. Study Population

We enrolled consecutive outpatients aged ≥ 75 years with T2DM who visited the outpatient department of Fujita Health University Hospital between June 2021 and June 2023. The age threshold of ≥75 years was selected because this group represents the “old-old” population according to the current Japanese classification, in which individuals aged 75–89 years are defined as “old” and those aged ≥90 years as “oldest-old.” Functional and metabolic decline, including anabolic resistance and reduced renal reserve, are markedly accelerated in this age range compared with individuals aged 65–74 years. Therefore, the study specifically targeted adults aged ≥ 75 years to evaluate nutritional interventions in those at highest risk for sarcopenia progression.

Participants were consecutively recruited from the outpatient clinics of the Department of Endocrinology, Diabetes and Metabolism at Fujita Health University Hospital (Aichi, Japan), a tertiary care institution providing comprehensive diabetes management. Eligible participants were community-dwelling adults aged ≥ 75 years with type 2 diabetes who attended the clinic for regular diabetes care or were referred for nutritional evaluation and guidance by their attending endocrinologists. Recruitment occurred between June 2021 and June 2023. The age threshold (≥75 years) was pre-specified to target the oldest-old segment in whom sarcopenia prevalence and adverse outcomes accelerate, CKD prevalence is substantially higher, and anabolic resistance is more pronounced, as detailed in the Introduction.

Individuals were excluded if they had (1) clinically evident dementia or psychiatric disease; (2) steroid use exceeding physiological replacement; (3) hepatic dysfunction (Child–Pugh ≥ 7) or renal impairment defined as serum creatinine ≥ 2.0 mg/dL; (4) symptomatic heart failure (NYHA ≥ II); (5) advanced diabetic complications prohibiting exercise therapy (e.g., nephropathy stage ≥ 3, proliferative retinopathy, or severe neuropathy); (6) systemic infection fulfilling SIRS criteria or severe trauma (AIS ≥ 4); (7) incurable malignancy; (8) implanted pacemaker or cardioverter-defibrillator; (9) central neuromuscular disease causing limb paralysis; or (10) any other condition deemed inappropriate by the investigators. Consequently, participants with preserved or mildly reduced renal function (predominantly CKD G1–G3) were enrolled to ensure the safety of high-protein nutritional guidance.

### 2.2. Study Designs and Ethical Considerations

This was a single-arm, exploratory, prospective, open-label interventional study aimed at evaluating the effects of individualized nutritional counseling on sarcopenia-related indicators in older adults with T2DM. The intervention consisted of non-invasive, standard-of-care dietary guidance delivered as part of routine clinical practice. This study was conducted in accordance with the Declaration of Helsinki and was approved by the Ethics Review Committee for Medical Research, Fujita Health University (project identification code HM23-351; approval date: 14 May 2021). This clinical trial was prospectively registered in the UMIN Clinical Trials Registry (UMIN-CTR) prior to participant enrollment (Registration ID: UMIN000044687; Date of registration: 28 June 2021). Written informed consent was obtained from all participants prior to inclusion in the study. The unit of assignment and analysis was the individual. All participants were allocated to the same intervention condition (nonrandomized, single-arm). Blinding of participants and dietitians was not feasible; laboratory assays were performed by technicians blinded to timepoints where applicable.

### 2.3. Intervention and Follow-Up Schedule

Registered dietitians provided individualized nutritional counseling at baseline, 6 months, and 12 months, targeting protein intake ≥ 1.0 g/kg/day and energy intake 25–35 kcal/kg/day. Protein intake targets were established according to the ESPEN recommendations for older adults (minimum target ≥ 1.0 g/kg ideal body weight [IBW]/day) and the Japanese Society of Nephrology’s 2019 statement on dietary management for non-dialysis CKD patients with sarcopenia or frailty [23].

Accordingly, individualized counselling targets were set as follows: 1.5 g/kg IBW/day for participants with CKD stage G1–G2 and 1.3 g/kg IBW/day for those with CKD stage G3. No participants in this study had CKD stage G4 or higher.

All protein targets were calculated based on IBW (22 × height^2^) and adjusted according to each participant’s renal function and nutritional condition. Registered dietitians provided individualized feedback at each visit to help maintain protein intake above the ESPEN minimum recommendation. The intervention was conducted in the outpatient clinics of the Department of Endocrinology, Diabetes and Metabolism, Fujita Health University Hospital, as part of routine diabetes care. All counseling sessions were delivered by nationally licensed registered dietitians who also held credentials as Certified Diabetes Educators and had more than 8 years of professional experience in clinical nutrition.

Each session lasted approximately 30 min and was provided at baseline, 6 months, and 12 months, focusing on individualized meal planning. The counseling targeted a protein intake of 1.3–1.5 g per kg of ideal body weight (IBW) per day, emphasizing diverse protein sources including meat, fish, eggs, dairy products, and legumes.

Dietary intake and adherence were evaluated at baseline, 6, 12, and 18 months using a validated Food Frequency Questionnaire based on food groups (FFQg). Nutrient intake was analyzed using Excel Eiyou-kun Ver. 9 (Kenpakusha, Tokyo, Japan), which estimates daily energy and nutrient intake based on standard portion sizes and frequency data, referencing the Standard Tables of Food Composition in Japan (2020 edition). Adequate calcium and vitamin D intake and moderate physical activity were encouraged as part of general lifestyle advice. However, no standardized monitoring or quantification of physical activity was conducted, as the study primarily focused on the effects of nutritional guidance. Participants were advised to maintain regular walking or light resistance exercises according to their usual daily activity levels. Clinical assessments were performed at baseline and every 6 months up to 18 months (visits 1–4). Adherence to nutritional counselling was monitored at each visit (baseline [V1], 6 months [V2], and 12 months [V3]) using a validated Food Frequency Questionnaire based on food groups (FFQg). Registered dietitians reviewed FFQg responses at every visit and provided individualized feedback to ensure that participants maintained protein and micronutrient intakes above target levels. The counselling targeted a protein intake of approximately 1.4 g/kg IBW/day and calcium and vitamin D intakes of ~640 mg/day and 8.5 µg/day, respectively. Nutritional guidance emphasized food-based improvement rather than pharmacologic supplementation. Adequacy of vitamin D intake was also evaluated by serum 25-hydroxyvitamin D [25(OH)D] measurements, excluding participants taking active vitamin D analogs (e.g., alfacalcidol, eldecalcitol). Paired *t*-tests were used to compare actual nutrient intakes with target values at each visit.

Baseline diabetes medications included glucagon-like peptide-1 receptor agonists (*n* = 16), insulin (*n* = 13), metformin (*n* = 17), dipeptidyl peptidase-4 inhibitors (*n* = 21), sodium–glucose cotransporter-2 inhibitors (*n* = 12), glinides (*n* = 19), alpha-glucosidase inhibitors (*n* = 21), sulfonylureas (*n* = 5), and pioglitazone (*n* = 7).

Because this was a clinical-practice-based study, changes in antidiabetic therapy were made at the discretion of the treating physicians, and no protocol-based restrictions were imposed.

Physical activity was encouraged as part of standard lifestyle advice; however, it was not quantified using a standardized questionnaire, which is acknowledged as a study limitation.

### 2.4. Outcomes and Assessments

The primary outcomes were skeletal muscle index (SMI), grip strength, and gait speed.

Sarcopenia was diagnosed according to the Asian Working Group for Sarcopenia (AWGS) 2019 consensus [24]. Participants were classified as having sarcopenia if they exhibited low muscle mass plus either low muscle strength or slow gait speed.

The diagnostic cutoffs were as follows:

- Low muscle mass: SMI < 7.0 kg/m^2^ for men and <5.7 kg/m^2^ for women, measured by bioelectrical impedance analysis (InBody 770; InBody Co., Ltd., Seoul, Republic of Korea).
- Low muscle strength: Grip strength < 28 kg for men and <18 kg for women, assessed using a digital handgrip dynamometer (Smedley Hand Dynamometer^®^; Saehan Corporation, Changwon, Republic of Korea). Each measurement was performed three times within a 5 min interval, and the maximum value was recorded.
- Slow gait speed: <1.0 m/s, evaluated using the 6 m usual gait speed test recommended by AWGS 2019.

These parameters were used to determine the prevalence of sarcopenia and its changes over the study period.

Secondary outcomes included serum AAs concentrations, renal function (eGFR based on creatinine, eGFRcreat; and eGFR based on cystatin C, eGFRcys), hepatic steatosis/fibrosis (controlled attenuation parameter, CAP [dB/m]; liver stiffness by elastography [kPa]), insulin resistance indices (TG/HDL-C ratio[Triglyceride/High-Density Lipoprotein Cholesterol ratio], TyG index[Triglyceride/Glucose index]), and endocrine/metabolic markers (glucagon, C-peptide [CPR], FGF21, hemoglobin A1c [HbA1c], free fatty acids [FFA]). CKD was defined as eGFRcreat < 60 mL/min/1.73 m^2^ [25]. Dietary intake was assessed using a validated Food Frequency Questionnaire based on food groups (FFQg), which is suitable for estimating nutrient intake in Japanese populations [26]. Nutritional counseling followed the 7th edition of the Food Exchange List for Dietary Therapy in Diabetes (Japan Diabetes Society) [27]. Fasting blood samples were collected in the morning. Glucagon was measured using a glucagon ELISA (Mercodia, Uppsala, Sweden) and FGF21 using an FGF21 ELISA (BioVendor, Brno, Czech Republic). Routine biochemistry was analyzed on an automated analyzer (LABOSPECT 008; Hitachi High-Tech, Tokyo, Japan). Serum AAs were quantified at four time points (baseline, 6, 12, 18 months) by high-performance liquid chromatography (HPLC). For graphical presentation and biological interpretation, amino acids were categorized into five groups:

1. Branched-chain amino acids (BCAAs): valine, leucine, isoleucine;
2. Aromatic amino acids (AAAs): phenylalanine, tyrosine, tryptophan;
3. Glucogenic amino acids: alanine, serine, glycine, glutamine, etc.;
4. Sulfur-containing amino acids: cysteine and methionine;
5. Others: amino acids not included in the above categories.

Hepatic steatosis and fibrosis were assessed by transient elastography (FibroScan; Echosens, Paris, France) to obtain CAP (dB/m) and liver stiffness (kPa). eGFRcys and eGFRcreat were calculated at baseline, 6, 12, and 18 months. TG/HDL-C ratio and TyG index were calculated at baseline and every 6 months. Linear mixed-effects models (LMMs) with time as a fixed effect and participant as a random intercept were used to estimate longitudinal trends; annualized slopes (β/year) were derived.

### 2.5. Statistical Analysis

Analyses were performed using JMP version 18.0.1 (SAS Institute Inc., Cary, NC, USA). Continuous data is presented as mean ± SD. Paired *t*-tests were used to assess changes versus baseline. We computed Pearson correlation coefficients between AAs (*n* = 24; including BCAAs and total AAs), SMI, grip strength, and gait speed; partial correlations were additionally adjusted for age and sex (sex coded 0 = male, 1 = female). To minimize the effect of sex-related differences in muscle parameters, sex-normalized indices were calculated as follows: the normalized skeletal muscle index (nSMI) was obtained by dividing the individual SMI by the sex-specific diagnostic cut-off defined by AWGS 2019 (7.0 kg/m^2^ for men and 5.7 kg/m^2^ for women), and the normalized grip strength (nGrip) was calculated by dividing grip strength by the respective sex-specific cut-off (28 kg for men and 18 kg for women). These normalized variables (ΔnSMI, ΔnGrip) were used in regression analyses to evaluate changes in muscle parameters. Stepwise multiple linear regression identified independent predictors of the three sarcopenia indices. Two-sided *p* < 0.05 was considered statistically significant. Because this study was designed as an exploratory, single-arm, pre–post intervention, all statistical tests were considered exploratory.

Accordingly, no correction for multiple comparisons was applied.

The findings should therefore be interpreted with caution and regarded as hypothesis-generating.

This exploratory study had no formal sample-size calculation; all eligible patients during June 2021–June 2023 (*n* = 44) were enrolled. Listwise deletion was used: no imputation.

### 2.6. Principal Component Analysis (PCA) of Δ AAs

For multivariate profiling, PCA was performed on 6-month changes in serum AAs concentrations. Each change was defined as ΔAAs = AAs 6M − AAs baseline (three-letter codes). The dataset for PCA comprised already standardized Δ values (mean 0, SD 1) for 22 AAs; no additional difference or standardization procedures were performed. To avoid imputation-related artifacts, participants with any missing value among the 22 standardized ΔAAs were excluded listwise (final *n* = 39 for PCA). PCA was conducted on the correlation structure (equivalently, the covariance of z-scored variables) using singular value decomposition without rotation; components were ordered by decreasing eigenvalues. Eleven components explained 90.38% of total variance. Scree plot, loading matrices, and component scores were used for interpretation.

## 3. Results

### 3.1. Participant Flow and Baseline Characteristics

**Figure 1:**
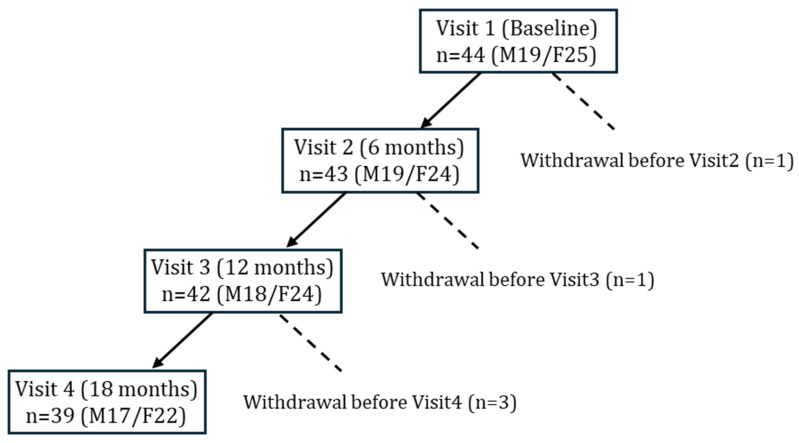
Flow of participants through the 18-month nutritional intervention study.

A total of 44 older adults with T2DM underwent baseline assessments at study entry (Figure 1). During follow-up, five participants were discontinued due to pancreatic cancer, prostate cancer, death, lower-limb amputation, or voluntary withdrawal. In accordance with the intention-to-treat principle, data obtained prior to withdrawal were included in the analysis. Ultimately, 39 participants completed the 18-month follow-up and were included in the longitudinal evaluation.

Forty-four older adults with T2DM were enrolled at baseline (Visit 1). Withdrawals occurred before Visit 2 (*n* = 1), Visit 3 (*n* = 1), and Visit 4 (*n* = 3) due to pancreatic cancer, voluntary discontinuation, death, prostate cancer, or lower limb amputation. Data from withdrawn participants was included in analyses when available. Final sample sizes were *n* = 43 at 6 months, *n* = 42 at 12 months, and *n* = 39 at 18 months.

Baseline characteristics of the 44 participants (19 men, 25 women) are summarized in Table 1. The mean age was 80.0 ± 3.8 years, without a sex difference. Men had significantly greater height, weight, total body water, protein mass, muscle mass, skeletal muscle mass, and SMI than women. Grip strength was higher in men, whereas gait speed did not differ by sex. Biochemically, men showed higher serum creatinine and lower eGFRcreat, consistent with higher muscle mass; uric acid was also higher in men. No sex differences were observed for aspartate aminotransferase (AST), alanine aminotransferase (ALT), gamma-glutamyl transferase (GGT), Fibrosis-4 index (FIB4 index), liver stiffness (elastography), or CAP. Glycemic markers (HbA1c, fasting glucose), endocrine markers (CPR, glucagon, FGF21), lipid profiles (triglycerides, low-density lipoprotein cholesterol[LDL-C], and high-density lipoprotein cholesterol [HDL-C]) were comparable between sexes. Overall, participants were functionally independent with relatively preserved muscle mass; however, lower grip strength in women suggested greater vulnerability to sarcopenia.

At baseline, participants retained at 18 months and those withdrawn did not differ in age, sex, SMI, gait speed, grip strength, or key laboratory values.

### 3.2. Nutritional Intake and Adherence

Table 2 summarizes baseline nutrient intakes and individualized dietary targets, showing that prescribed protein targets exceeded actual baseline intake levels. The mean prescribed protein intake was 1.41 ± 0.23 g/kg IBW at V1, 1.38 ± 0.16 g/kg IBW at V2, and 1.41 ± 0.14 g/kg IBW at V3. Corresponding actual protein intakes were 1.26 ± 0.37, 1.24 ± 0.42, 1.23 ± 0.44, and 1.23 ± 0.44 g/kg IBW at V1, V2, V3, and V4, respectively. At each visit, individualized targets were intentionally set above the participants’ current intake levels to encourage gradual dietary improvement, and this relationship between prescribed and achieved intake remained consistent across visits (all *p* < 0.05). Although the FFQg has been validated in Japanese populations, its 1-week reference period may not fully capture long-term habitual intake, particularly in very old adults whose diets vary seasonally or with health fluctuations. However, repeated FFQg assessments at each visit, combined with dietitian feedback, enabled ongoing monitoring and adjustment, thereby supporting the reliability of the intervention and confirming that protein intake was progressively reinforced throughout the 18-month period. The target intakes for vitamin D and calcium were 8.5 µg/day and ~640 mg/day, respectively.

Mean actual vitamin D intake was 7.7 ± 4.0 µg/day at V1, 7.2 ± 4.0 µg/day at V2, 7.0 ± 4.1 µg/day at V3, and 7.0 ± 4.1 µg/day at V4, showing no significant longitudinal changes (V1 vs. V2: *p* = 0.155; V1 vs. V3: *p* = 0.069; V1 vs. V4: *p* = 0.163).

Mean calcium intake was 553 ± 206 mg/day at V1, 573 ± 191 mg/day at V2, 535 ± 196 mg/day at V3, and 543 ± 241 mg/day at V4, also with no significant changes (all *p* > 0.1). Serum 25(OH)D concentrations (excluding vitamin D supplement users) were 17.7 ± 6.1 ng/mL at V1, 17.1 ± 6.9 ng/mL at V2, 18.8 ± 6.5 ng/mL at V3, and 18.4 ± 6.9 ng/mL at V4. A significant increase was observed at V3 compared with V1 (*p* = 0.015), while other intervals showed no significant differences (*p* > 0.26). The composition of protein sources (legumes, fish and seafood, meat, eggs, and dairy products) remained largely unchanged throughout the intervention, except for a transient increase in dairy product intake at 6 months (Supplementary Table S2).

### 3.3. Changes in Sarcopenia-Related Indices, Organ/Metabolic Parameters, and Metabolic Hormones

**Figure 2:**
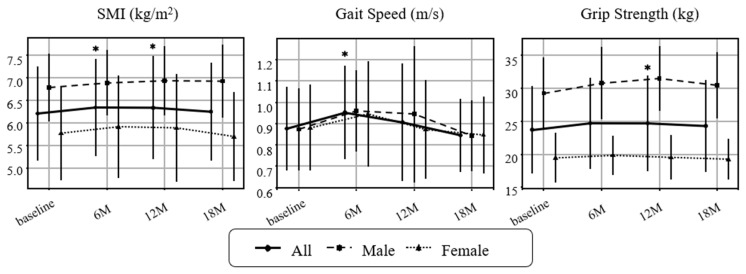
Changes in sarcopenia-related indicators over time. Longitudinal changes in skeletal mass index (SMI), gait speed, and grip strength are shown for all participants, as males and females. Values represent mean ± SD. Paired *t*-tests were performed between each post-intervention timepoint and baseline. Asterisks (*) indicate *p* < 0.05. Missing values were not imputed for analysis. Sample sizes: baseline *n* = 44 (M19/F25), 6 months *n* = 43 (M19/F24), 12 months *n* = 42 (M18/F24), 18 months *n* = 39 (M17/F22).

The primary outcomes were changes in sarcopenia-related indices, organ/metabolic parameters, and metabolic hormones. The prevalence of sarcopenia (AWGS 2019 criteria [24]) declined from 22/44 (50.0%) at baseline (11 men, 11 women) to 14/44 (31.8%) at 6 months, 15/42 (35.7%) at 12 months, and 17/39 (43.6%) at 18 months; the proportion at 6 months was significantly lower than baseline. Physical function and muscle-related outcomes improved (Figure 2). SMI increased significantly at 6 and 12 months, grip strength trended upward at 6 months and reached significance at 12 months, and gait speed improved at 6 months. None of these indices showed significant deterioration during follow-up. Subgroup analyses suggested larger gains in SMI and grip strength among men than women (Exploratory).

**Figure 3:**
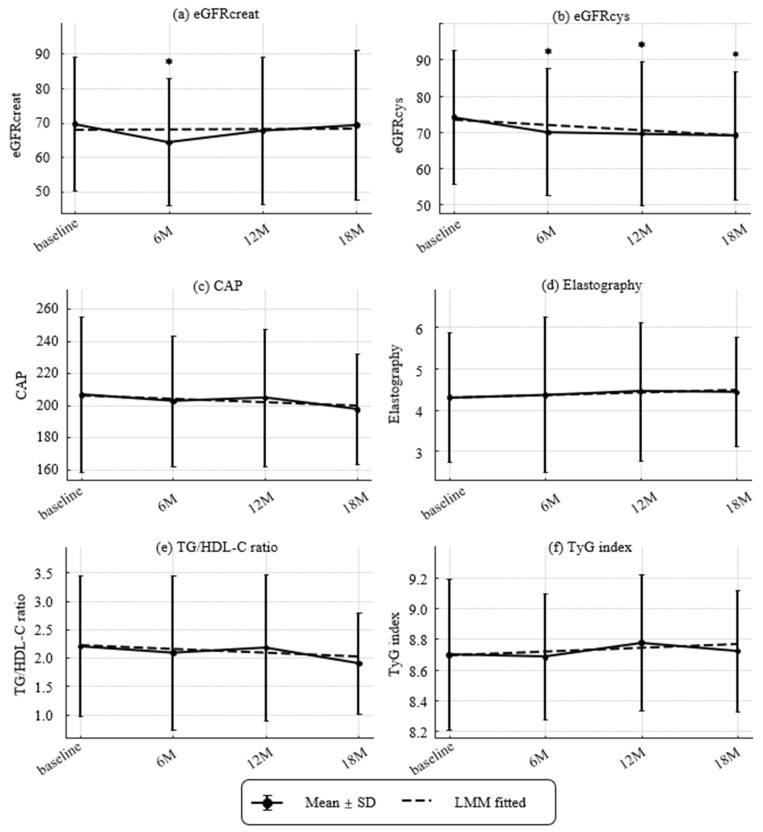
Longitudinal Changes in Renal and Metabolic Parameters. (**a**) Estimated glomerular filtration ratio (eGFR) based on creatinine (eGFRcreat) (mL/min/1.73 m^2^), (**b**) eGFR based on cystatin C (eGFRcys) (mL/min/1.73 m^2^), (**c**) Controlled attenuated parameter (CAP) (dB/m), (**d**) elastography (kPa), (**e**) TG/HDL-C ratio, and (**f**) TyG index. Values represent mean ± SD. Asterisks denote significant differences compared with baseline by paired *t*-tests (* p < 0.05). Dashed lines indicate fitted trends estimated by linear mixed-effects models (LMMs). Estimated annual slopes (95% CI) were as follows:(**a**) eGFRcreat: +0.18/year (−1.96 to +2.32, *p* = 0.8674); (**b**) eGFRcys: −2.97/year (−4.62 to −1.31, *p* = 0.0004); (**c**) CAP: −3.89/year (−13.46 to +5.67, *p* = 0.4249); (**d**) Elastography: +0.13/year (−0.21 to +0.47, *p* = 0.4559); (**e**) TG/HDL-C ratio: −0.14/year (−0.34 to +0.06, *p* = 0.1812); (**f**) TyG index: +0.05/year (−0.04 to +0.13, *p* = 0.2606).

**Figure 4:**
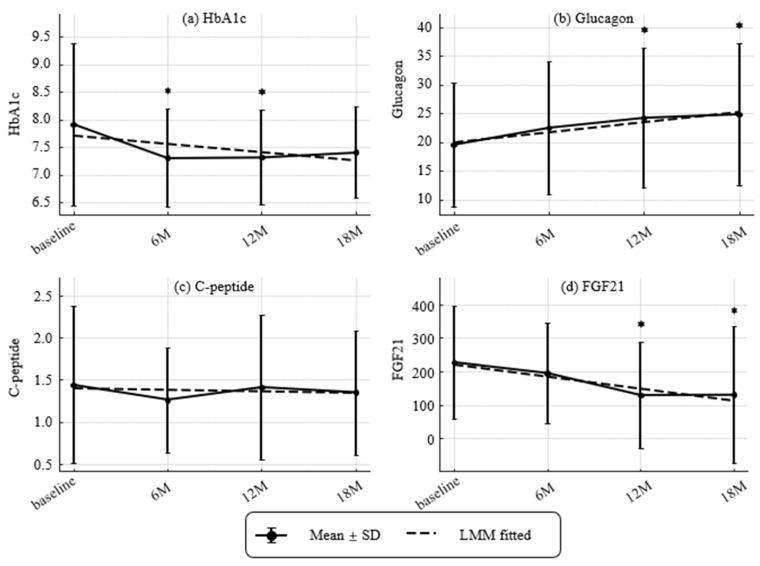
Longitudinal changes in metabolic markers and hormones (HbA1c, glucagon, CPR, and Fibroblast Growth Factor 21 [FGF21]). (**a**) HbA1c (NGSP, %), (**b**) glucagon (pg/mL), (**c**) C-peptide (CPR) (ng/mL) and (**d**) Fibroblast growth factor 21(FGF21) (pg/mL). Values represent mean ± SD. Asterisks indicate statistically significant differences from baseline by paired *t*-tests (* p < 0.05). Dashed lines indicate trends estimated by linear mixed-effects models (LMMs). Estimated annual slopes (95% CI) were:(**a**) HbA1c: −0.26%/year; (**b**) Glucagon: +3.49 pg/mL/year; (**c**) CPR: −0.03 ng/mL/year; (**d**) FGF21: −72.94 pg/mL/year. Missing values were not imputed.

Longitudinal changes in renal, hepatic, and metabolic parameters are shown in Figure 3 and Figure 4, which illustrate dynamic trajectories over 18 months. Figure 3 and Figure 4 present longitudinal data, while Table 1 summarizes baseline characteristics.

Renal, hepatic, and metabolic trajectories are shown in Figure 3. The prevalence of CKD (eGFRcreat < 60 mL/min/1.73 m^2^ [25]) was 14/44 (31.8%) at baseline, 19/43 (44.2%) at 6 months, 13/42 (31.0%) at 12 months, and 13/39 (33.3%) at 18 months, with no significant change over time. Over 18 months, eGFRcys declined significantly with an annualized slope of −2.97 mL/min/1.73 m^2^ per year (*p* = 0.00042), while eGFRcreat showed no significant change (+0.18 mL/min/1.73 m^2^ per year; *p* = 0.867). Hepatic measures were stable overall. ΔCAP was −3.89 dB/m per year (*p* = 0.422) and liver stiffness was +0.13 kPa per year (*p* = 0.454). Atherogenic/metabolic indices showed no linear trends. ΔTG/HDL-C ratio was −0.14 per year (*p* = 0.179) and ΔTyG index was +0.049 per year (*p* = 0.260).

Temporal patterns of HbA1c, glucagon, CPR, and FGF21 are presented in Figure 4. HbA1c decreased at 6 and 12 months (modeled slope −0.26% per year). Glucagon increased at 12 and 18 months (modeled slope +3.49 pg/mL per year). CPR remained stable (slope −0.03 ng/mL per year). FGF21 declined at 12 and 18 months (slope −72.94 pg/mL per year).

### 3.4. Baseline Associations of AAs and Hormones with Sarcopenia-Related Indices

#### Cross-Sectional Associations at Baseline Are Shown in Table 3

In univariate analyses, SMI correlated positively with total AAs, especially BCAAs (valine [Val], leucine [Leu], isoleucine [Ile]), ornithine (Orn), glutamic acid (Glu), and proline (Pro).

After adjustment for age and sex, significant associations were found for cysteine (Cys), Orn, and Glu.

Grip strength correlated positively with Val and Leu in unadjusted models; these associations attenuated and were not significant after adjustment. By contrast, gait speed showed negative associations with Ile, alanine (Ala), and total AAs. After adjustment for age and sex, Leu, Ile, Ala, BCAAs, and total AAs showed inverse associations with gait speed.

As shown in Table 3, the most notable associations were positive correlations of SMI with Orn, Cys, and Glu, and negative correlations of gait speed with Leu, Ile, and Ala. These results suggest that Orn, Cys, and Glu may play a potential role in supporting muscle protein anabolism, whereas the inverse relationships between BCAAs and Ala with gait speed may reflect accumulation of amino acids under reduced utilization or catabolic stress.

**Table 3:** Correlations between serum amino acids (AAs) levels and sarcopenia indices.

AAs	Univariate Analysis				Multivariate Analysis	
	Non-Adjusted		Adjusted for Age and Sex			
	r	P (r)	Partial_r	P (Partial_r)	Beta (95% CI)	P (Beta)
(a) Relationship between skeletal mass index (SMI) and amino acids (AAs)						
Val	**0.31**	0.039	0.07	0.633	0.00 (−0.01–0.01)	0.642
Leu	**0.36**	0.016	0.17	0.269	0.01 (−0.01–0.02)	0.281
Ile	**0.35**	0.021	0.16	0.293	0.01 (−0.01–0.02)	0.305
Tyr	−0.16	0.290	−0.07	0.642	−0.01 (−0.03–0.02)	0.650
Phe	0.26	0.089	0.23	0.137	0.02 (−0.01–0.05)	0.146
Trp	0.04	0.787	−0.09	0.566	−0.01 (−0.04–0.02)	0.576
Met	0.2	0.191	0.2	0.187	0.04 (−0.02–0.09)	0.198
Cys	0.28	0.066	**0.36**	0.018	**0.03 (0.00–0.05)**	0.021
Arg	−0.12	0.454	0.0	1.000	0.00 (−0.01–0.01)	1.000
Orn	**0.41**	0.005	**0.36**	0.015	**0.02 (0.00–0.03)**	0.018
Cit	0.16	0.304	0.22	0.155	0.02 (−0.01–0.04)	0.166
Ala	0.24	0.113	0.26	0.085	0.00 (−0.00–0.01)	0.093
Gln	0.02	0.881	0.08	0.619	0.00 (−0.00–0.00)	0.628
Glu	**0.41**	0.005	**0.35**	0.019	**0.02 (0.00–0.03)**	0.022
Asp	0.04	0.815	−0.06	0.723	−0.06 (−0.43–0.30)	0.730
Asn	0.15	0.345	0.14	0.366	0.01 (−0.01–0.04)	0.378
Ser	−0.2	0.200	−0.19	0.211	−0.01 (−0.02–0.00)	0.222
Gly	0.1	0.519	**0.3**	0.047	0.01 (−0.00–0.01)	0.053
His	0.23	0.138	0.18	0.239	0.02 (−0.01–0.04)	0.251
Thr	0.18	0.236	0.19	0.217	0.01 (−0.00–0.02)	0.228
Lys	0.09	0.565	0.04	0.801	0.00 (−0.01–0.01)	0.806
Pro	**0.37**	0.013	0.21	0.162	0.00 (−0.00–0.01)	0.172
BCAAs	**0.35**	0.020	0.13	0.389	0.00 (−0.00–0.01)	0.400
Total AAs	**0.3**	0.049	0.26	0.085	0.00 (−0.00–0.00)	0.093
(b) Relationship between Grip strength and amino acids (AAs)						
Val	**0.41**	0.005	0.06	0.710	0.01 (−0.03–0.05)	0.717
Leu	**0.38**	0.012	0.06	0.707	0.01 (−0.04–0.06)	0.714
Ile	0.27	0.078	−0.1	0.514	−0.02 (−0.10–0.05)	0.524
Tyr	−0.26	0.086	−0.17	0.273	−0.06 (−0.17–0.05)	0.284
Phe	0.08	0.624	−0.03	0.831	−0.02 (−0.17–0.14)	0.835
Trp	0.03	0.836	−0.22	0.154	−0.11 (−0.26–0.05)	0.165
Met	−0.01	0.964	−0.07	0.639	−0.06 (−0.33–0.21)	0.647
Cys	−0.07	0.667	−0.06	0.706	−0.02 (−0.14–0.10)	0.713
Arg	−0.27	0.072	−0.16	0.300	−0.03 (−0.09–0.03)	0.312
Orn	0.09	0.543	−0.12	0.428	−0.02 (−0.09–0.04)	0.439
Cit	−0.08	0.601	−0.1	0.519	−0.04 (−0.15–0.08)	0.529
Ala	0.0	0.997	−0.04	0.819	−0.00 (−0.02–0.01)	0.823
Gln	−0.14	0.354	−0.12	0.449	−0.01 (−0.02–0.01)	0.461
Glu	0.23	0.135	0.07	0.637	0.02 (−0.06–0.09)	0.645
Asp	0.03	0.854	−0.16	0.309	−0.87 (−2.64–0.89)	0.322
Asn	0.01	0.958	−0.04	0.778	−0.02 (−0.15–0.11)	0.784
Ser	−0.12	0.447	−0.07	0.660	−0.01 (−0.07–0.05)	0.668
Gly	−0.24	0.112	−0.03	0.840	−0.00 (−0.03–0.02)	0.843
His	0.15	0.328	0.06	0.693	0.03 (−0.11–0.17)	0.700
Thr	−0.05	0.763	−0.11	0.474	−0.02 (−0.06–0.03)	0.484
Lys	0.06	0.699	−0.03	0.856	−0.00 (−0.05–0.04)	0.859
Pro	0.25	0.096	−0.08	0.600	−0.01 (−0.04–0.02)	0.609
BCAAs	**0.39**	0.010	0.02	0.887	0.00 (−0.02–0.02)	0.890
Total AAs	0.06	0.692	−0.08	0.626	−0.00 (−0.00–0.00)	0.634
(c) Relationship between Gait speed and amino acids (AAs)						
Val	−0.21	0.176	−0.29	0.060	−0.00 (−0.00–0.00)	0.067
Leu	−0.29	0.064	**−0.37**	0.015	**−0.00 (−0.01–0.00)**	0.017
Ile	**−0.33**	0.032	**−0.4**	0.009	**−0.00 (−0.01–0.00)**	0.011
Tyr	−0.09	0.554	−0.09	0.553	−0.00 (−0.01–0.00)	0.564
Phe	−0.25	0.111	−0.26	0.092	−0.01 (−0.01–0.00)	0.100
Trp	−0.14	0.392	−0.17	0.290	−0.00 (−0.01–0.00)	0.303
Met	−0.24	0.118	−0.25	0.107	−0.01 (−0.02–0.00)	0.117
Cys	−0.24	0.127	−0.22	0.156	−0.00 (−0.01–0.00)	0.167
Arg	−0.1	0.525	−0.09	0.551	−0.00 (−0.00–0.00)	0.562
Orn	−0.19	0.223	−0.19	0.226	−0.00 (−0.00–0.00)	0.238
Cit	−0.07	0.659	−0.03	0.840	−0.00 (−0.01–0.01)	0.845
Ala	**−0.34**	0.029	**−0.38**	0.014	**−0.00 (−0.00–0.00)**	0.017
Gln	−0.17	0.296	−0.18	0.248	−0.00 (−0.00–0.00)	0.260
Glu	0.02	0.908	−0.02	0.906	−0.00 (−0.00–0.00)	0.909
Asp	−0.15	0.370	−0.16	0.338	−0.04 (−0.11–0.04)	0.351
Asn	−0.16	0.312	−0.17	0.272	−0.00 (−0.01–0.00)	0.285
Ser	−0.03	0.862	−0.09	0.559	−0.00 (−0.00–0.00)	0.569
Gly	−0.02	0.915	−0.04	0.794	−0.00 (−0.00–0.00)	0.799
His	−0.12	0.447	−0.11	0.472	−0.00 (−0.01–0.00)	0.483
Thr	−0.19	0.224	−0.22	0.156	−0.00 (−0.00–0.00)	0.167
Lys	−0.21	0.175	−0.26	0.094	−0.00 (−0.00–0.00)	0.102
Pro	−0.19	0.217	−0.24	0.125	−0.00 (−0.00–0.00)	0.135
BCAAs	−0.28	0.076	**−0.37**	0.017	**−0.00 (−0.00–0.00)**	0.021
Total AAs	**−0.31**	0.043	**−0.36**	0.021	**−0.00 (−0.00–0.00)**	0.025

Pearson’s correlation coefficients (r), partial correlation coefficients adjusted for age and sex (partial r), and standardized regression coefficients (β) with 95% confidence intervals (CI) were calculated for each of the AAs. Sex was coded as 0 (male) and 1 (female). Significant correlations (*p* < 0.05) are highlighted in bold. Abbreviations: Arg, arginine; Asn, asparagine; Asp, aspartic acid; BCAAs, branched chain amino acids; Cit, citrulline; Gln, glutamine; Gly, glycine; His, histidine; Lys, lysine; Met, methionine; Phe, phenylalanine; Ser, serine; Thr, threonine; Trp, tryptophan; Tyr, tyrosine.

These findings are consistent with previous metabolomic studies linking altered amino acid profiles to impaired muscle function in older adults [28].

We next explored associations of hormonal biomarkers with clinical/functional variables (Table 4). CPR correlated positively with BMI and fat mass, SMI, serum creatinine, and cystatin C, and negatively with eGFRcreat, eGFRcys, and HDL-C after adjustment for age and sex. CPR also correlated positively with ALT, γ-GTP, triglycerides, and FGF21. Glucagon showed no associations with any parameters in the adjusted model. FGF21 correlated positively with fat mass, cystatin C, and triglycerides, and negatively with gait speed, and grip strength.

### 3.5. Longitudinal Changes in AAs

**Figure 5:**
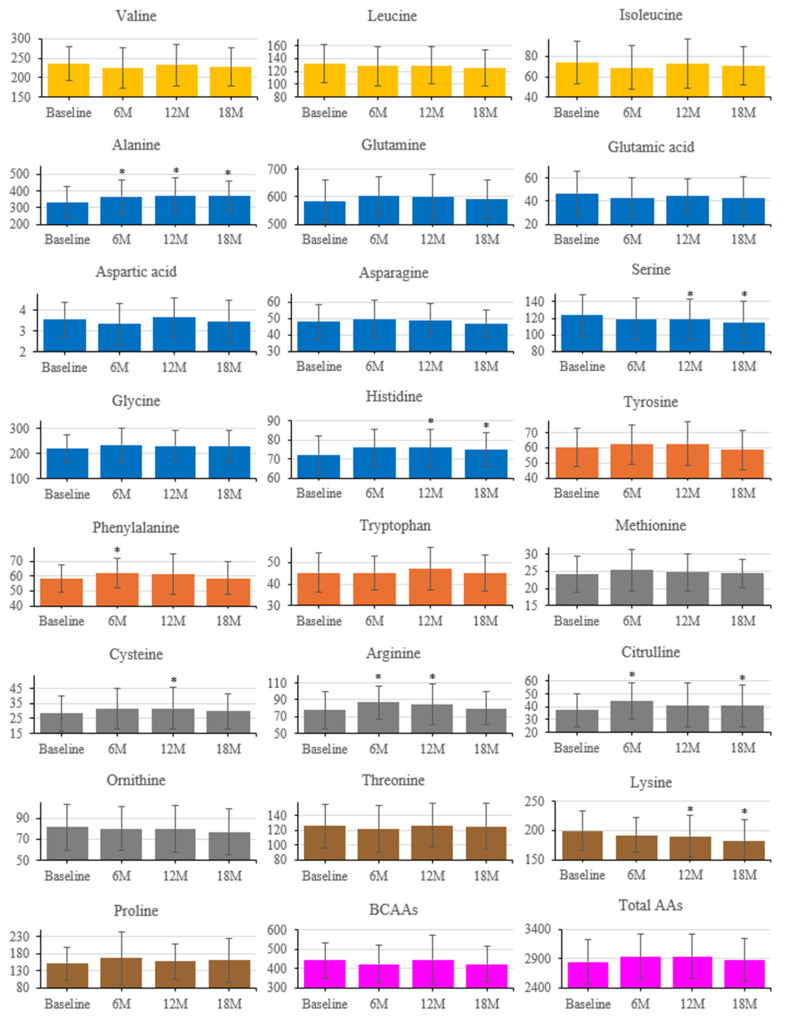
Longitudinal changes in serum amino acid concentrations after intervention. Amino acids are grouped by metabolic class (Orange, BCAAs; Dark orange, aromatic; Blue, glucogenic; Gray, sulfur-containing; Yellow Ochre, others; purple, BCAAs and Total AAs). Error bars indicate SD; * *p* < 0.05 vs. baseline (paired *t*-test).

Longitudinal changes in serum AAs are shown in Figure 5 (baseline, 6, 12, 18 months). Several AAs increased from baseline: Phe at 6 months; Cys at 12 months; Arg at 6 and 12 months; Cit at 6 and 18 months; Ala at 6, 12, and 18 months; and his at 12 and 18 months. Ser and Lys decreased from baseline at 12 and 18 months. Total AAs and the sum of BCAAs did not show significant longitudinal changes.

### 3.6. PCA and Multivariable Modeling of Changes in Sarcopenia Outcomes (Exploratory)

**Figure 6:**
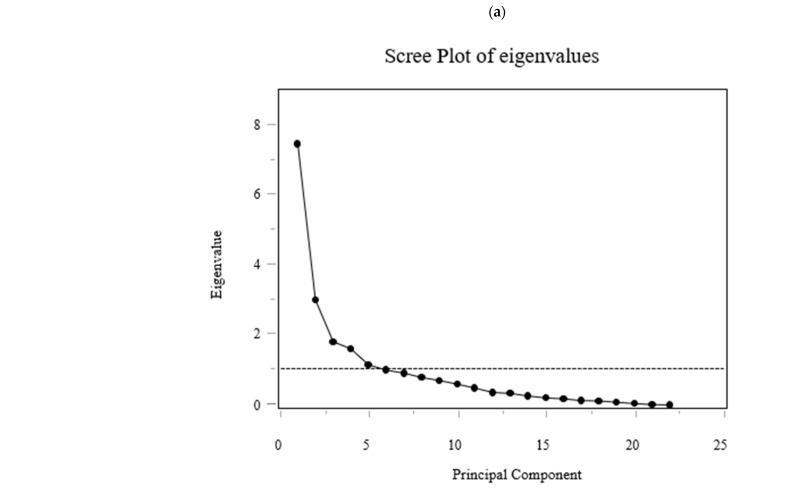

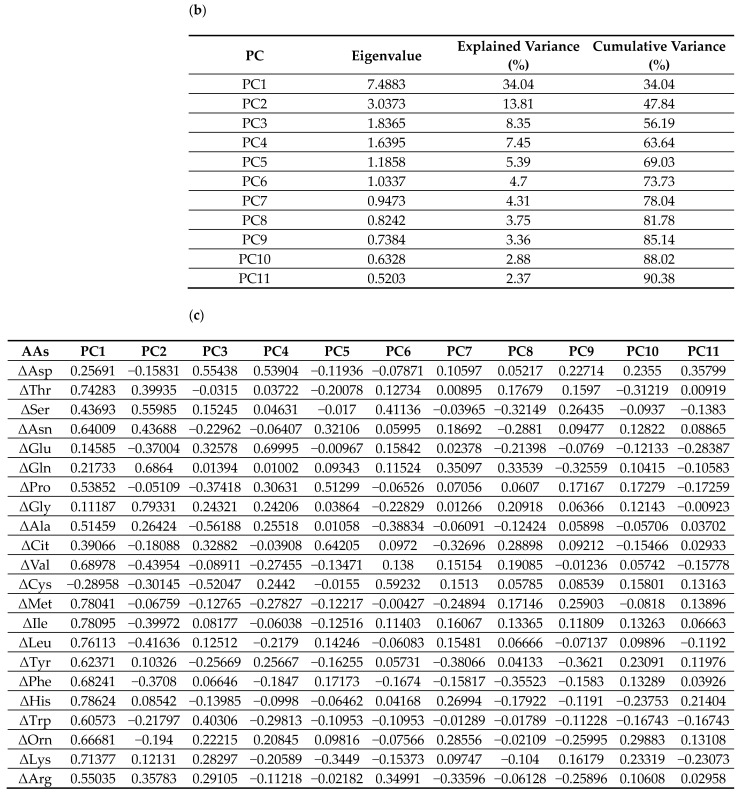
(**a**) Scree plot of eigenvalues; (**b**) Table showing eigenvalues, explained variance, and cumulative variance for the first 11 components; (**c**) Loading matrix for amino acids (AAs) (ΔAAs) on PC1–PC11. Principal component analysis (PCA) of changes in serum amino acid concentrations (Δ from baseline to 6 months). The PCA summarizes coordinated multivariate patterns among amino acids, complementing the individual trends shown in Figure 5. Multiple linear regression analyses were performed to identify predictors of changes in sarcopenia-related outcomes. The dependent variables were changes in sex-normalized SMI (ΔnSMI) and grip strength (ΔnGrip). Sex normalization was conducted by dividing each value by the sex-specific diagnostic thresholds (SMI: 7.0 kg/m^2^ for men, 5.7 kg/m^2^ for women; grip strength: 28 kg for men, 18 kg for women). Multiple regression analyses identified glucagon, CPR, and amino acid component scores as significant predictors of changes in sex-normalized skeletal muscle index and grip strength (Table 5). Full model details are available in Supplementary Table S1.

While Figure 5 displays individual amino acid trajectories, Figure 6 summarizes coordinated multivariate patterns derived from the same dataset using principal component analysis (PCA).

PCA on standardized 6-month Δ AAs identified 11 components explaining 90.38% of total variance (Figure 6). PC1 and PC2 explained 34.0% and 13.8% (cumulative 47.8%). Loadings on PC1 were largest for BCAAs (ΔVal, ΔLeu, ΔIle), aromatic amino acids (aromatic AAs; ΔThr, ΔPhe, ΔTrp), and essential AAs (ΔLys, ΔMet, ΔHis). PC2 contrasted glucogenic/non-essential ΔAAs (ΔGly, ΔSer, ΔGln; with ΔAla positive) versus BCAAs/aromatic AAs (negative). PC3 opposed urea-cycle/acidic/basic ΔAAs (ΔAsp, ΔGlu, ΔCit, ΔOrn, ΔArg, ΔLys; negative) to ΔAla/ΔCys/ΔPro/ΔTrp (positive). The loading tables (PC1–PC11) are shown in Figure 6 (Exploratory).

The regression model for ΔnSMI was statistically significant. ΔGlucagon was positively associated with ΔnSMI, whereas ΔCPR and PC8 were negatively associated (standardized β ≈ +0.41, −0.41, and −0.42; *p* ≈ 0.020, 0.022, and 0.0045, respectively). Inspection of PC8 loadings indicated a contrast between a positive side enriched for Gln/Gly (gluconeogenic/one-carbon) and Cit (urea-cycle), together with selected essential/BCAAs/aromatic AAs signals (Val, Met, Thr, Trp), versus a negative side marked by Phe (aromatic AAs), Ser/Asn (gluconeogenic), Glu (acidic), and His (basic), consistent with a nitrogen-disposal–oriented routing that blunts net muscle accretion. The regression model for ΔnGrip was also significant. ΔGlucagon was negatively associated with ΔnGrip. In addition, PC1 and PC7 were also negatively associated with ΔnGrip. Six-month increases in the concentrations of BCAAs (Val, Leu, Ile), aromatic AAs (Tyr, Phe, Trp), Thr, Ser, Asn, Pro, Ala, Met, His, Orn, Lys, and Arg were associated with reduced nGrip. Changes in gait speed could not be modeled adequately, likely due to multicollinearity or limited variance.

**Table 5:** Multiple linear regression analysis for changes in sarcopenia-related indices.

Dependent Variable	Independent Variable	β	Std. β	*p*-Value
ΔnSMI	ΔGlucagon	+0.16	+0.41	0.020 *
	ΔCPR	−2.81	−0.41	0.022 *
	PC8	−2.46	−0.42	0.005 *
ΔnGrip	ΔGlucagon	−0.40	−0.09	0.028 *
	PC1	−2.09	−0.36	0.013 *
	PC7	−5.28	−0.39	0.026 *

Abbreviations: SMI, skeletal muscle index; nSMI, sex-normalized SMI; nGrip, sex-normalized grip strength; CPR, C-peptide; PC, principal component score derived from changes in 22 amino acids. Note: Only significant predictors are shown (* *p* < 0.05). Full regression models are presented in Supplementary Table S1.

### 3.7. Adverse Events

No study-related adverse events or unintended effects occurred during the 18-month follow-up.

## 4. Discussion

Primary outcomes focused on changes in muscle-related parameters and renal safety, while exploratory analyses identified potential metabolic correlates of these changes.

This discussion is organized into four main sections:

- Effects of the intervention on sarcopenia-related parameters;
- Renal and hepatic safety;
- Metabolic mechanisms involving hormones and amino acids;
- Clinical implications for adults aged ≥ 75 years with T2DM.

### 4.1. Effects of the Intervention on Sarcopenia-Related Parameters

In the present study, we demonstrate that provision of nutritional guidance interventions every six months for 18 months to individuals aged 75 years or older with T2DM resulted in a transient increase in gait speed and sustained increases in muscle mass and grip strength without worsening renal function. Although some changes might reflect age-related physiological processes inherent to this advanced-age population, the observed improvements suggest that individualized nutritional guidance may help attenuate such declines.

In addition, although the 18-month follow-up provided valuable medium-term data, longer observation will be required to determine the sustained renal and hepatic effects of high-protein nutritional guidance.

### 4.2. Renal and Hepatic Safety

Although eGFRcys showed a modest but statistically significant decline (−2.97 mL/min/1.73 m^2^/year), this rate was markedly smaller than that reported in diabetic nephropathy cohorts, such as the CREDENCE placebo group (−4.7 mL/min/1.73 m^2^/year) [29].The decrease was observed mainly during the first 6 months and subsequently plateaued, while eGFRcreat and CKD staging remained stable. This pattern may reflect a benign hemodynamic adaptation similar to the “initial dip” seen with SGLT2 inhibitors, rather than progressive renal injury. Therefore, despite a transient decline in eGFRcys, no clinically significant deterioration of renal function was observed during 18 months of high-protein nutritional guidance in older adults with type 2 diabetes. The dietary intervention effectively established and maintained protein intake targets above baseline levels (Table 2), confirming adherence to individualized nutritional guidance. Exploratory regression analyses further indicated that glucagon and amino acid metabolism were associated with improvements in muscle parameters (Table 5 and Supplementary Table S1). Furthermore, we also found that changes in SMI were positively correlated with the changes in plasma glucagon levels and negatively correlated with the changes in plasma CPR levels, whereas changes in grip strength were inversely correlated with the changes in plasma glucagon. Muscle mass decreases with age, but it is known that lower-limb muscle strength declines in older adults before muscle mass decreases [30]. Particularly in individuals with diabetes, the decline in muscle strength and muscle mass is pronounced due to insufficient insulin secretion and action [31]. AAs contributes to muscle synthesis alongside insulin [32]. Previous studies have reported a positive correlation between protein intake and muscle mass or strength in both elderly individuals with and without diabetes [33,34]. However, there are few clinical studies that have examined these muscle parameters after the intervention of a high-protein diet in elderly individuals. Recently, it was reported that providing high-protein intake at 1.5 g/kg/day for 6 months to hospitalized individuals aged 75 years or older with cancer, fractures, pneumonia, or urinary tract infections resulted in increased grip strength in comparison with individuals receiving standard protein intake at 1.0 g/kg/day [8]. In the present study, we conducted an 18-month intervention providing individualized high-protein dietary guidance with targets of 1.5 g/kg IBW/day for CKD stage G1–G2, 1.3 g/kg IBW/day for CKD stage G3, and a minimum of ≥1.0 g/kg IBW/day in accordance with the ESPEN recommendations for older adults.

The achieved protein intakes (1.26 ± 0.37 to 1.23 ± 0.44 g/kg IBW across visits) confirmed that participants maintained intake levels above the recommended minimum throughout the 18-month intervention.

Temporal heterogeneity of responses was evident across sarcopenia indices. Gait speed improved early (6 months), consisting of rapid neuromuscular adaptations and task familiarity, but did not remain statistically different thereafter. SMI increased at 6 and 12 months, indicating structural accretion during the mid-phase of the intervention, yet the effect was not sustained at 18 months, possibly reflecting waning adherence, reduced sample size, or biological plateauing in very old adults. Grip strength peaked at 12 months only, suggesting delayed translation of mass accretion into strength with subsequent attenuation. Collectively, these findings argue for booster strategies (e.g., supervised resistance exercise blocks, adherence reinforcement, protein timing/quality optimization) to maintain gains beyond 12 months, particularly in 75 years and above populations with multimorbidity. Lower-limb motor function is determined by multiple factors, including balance, posture, vision, pain, and cognitive function [35,36]. It is possible that the improvement in lower-limb muscle strength was temporary due to impairments in multiple factors in the adults aged 75 years or older in our study.

### 4.3. Metabolic Mechanisms Involving Hormones and Amino Acids

An important aspect of our study is the relationship between AAs and hormonal profiles with changes in muscle parameters. Some AAs promote glucagon secretion from pancreatic alpha cells [37], and glucagon regulates the metabolism of AAs in the liver [17]. Various animal models deficient in glucagon action show hyperaminoacidemia due to impaired glucagon signaling in the liver [18]. Glucagon receptor-deficient mice show increased lean mass [38], and glucagon-deficient mice display increased lean mass and grip strength concomitant with a slow-to-fast transition in type II fibers of skeletal muscle [39], likely due to hyperaminoacidemia. These findings led us to investigate the relationship between plasma glucagon and AAs levels and muscle parameters. At baseline, SMI correlated positively with Orn, Cys, and Glu, whereas gait speed correlated negatively with BCAAs and Ala, suggesting differential amino acid utilization between anabolic and catabolic states. In univariate analyses, baseline SMI and grip strength showed a positive correlation with plasma BCAAs levels, as previously reported [40], but this correlation was not observed after adjusting for age and sex in our participants aged ≥ 75 years with T2DM. On the other hand, baseline gait speed displayed a negative correlation with plasma BCAAs, Ala, and total AAs levels after adjusting for age and sex. This suggests that AAs may play distinct roles in regulating upper- and lower-limb muscles. However, it remains to be investigated whether increased muscle breakdown in the lower limbs compared to the upper limbs leads to a decline in lower-limb strength concomitant with elevated plasma AAs concentrations, including Ala [41].

Next, we examined the changes in plasma AAs and glucagon levels in our participants aged ≥ 75 years with T2DM. Although plasma total AAs and BCAAs remained unchanged over time, plasma Phe, Cys, Arg, Cit, Ala, His, and glucagon levels increased after the intervention. AAs such as Arg and Ala stimulate glucagon secretion in rodents and humans [42,43]. Thus, the increase in plasma glucagon concentration under fasting conditions may be caused by the rise in these AAs.

We further investigated whether changes in plasma AAs and glucagon influenced changes in SMI and muscle strength during the first 6 months of dietary guidance, when improvements in SMI and muscle strength were most pronounced. Longitudinal changes in plasma AAs were associated with changes in SMI and grip strength. Interestingly, plasma Ser and Lys concentrations decreased following dietary instruction, and their decline was paradoxically associated with improvements in grip strength. This contrasts with recent reports linking low Ser and Lys to early sarcopenia [28]. This discrepancy may be explained by differences in study duration, participant age, or difference in the proportion of patients with diabetes. 

Because those findings were derived from cross-sectional comparisons, they may reflect existing metabolic characteristics of sarcopenic individuals rather than causal determinants.

In our longitudinal analysis, decreases in Ser and Lys were observed alongside improvements in grip strength, indicating temporary utilization of these amino acids within adaptive metabolic pathways during muscle strengthening.

Thus, the apparent difference from previous reports is likely due to differences in study design (cross-sectional vs. longitudinal) and interpretation, rather than a true contradiction. Which factors regulate plasma Ser and Lys concentrations, including insulin resistance, remain unclear. While BCAAs are well-known contributors to muscle mass and strength [44], the effects of Ser and Lys on muscle health in adults aged ≥ 75 years with T2DM require further investigation.

On the other hand, the change in plasma glucagon levels was negatively associated with the change in grip strength. These results are consistent with mouse studies showing that blockade of glucagon action enhances grip strength [39]. Interestingly, changes in glucagon were positively associated, and changes in CPR negatively associated, with changes in SMI in our subjects. HbA1c improved despite no change in plasma CPR levels after the intervention, suggesting that insulin sensitivity may have been enhanced. Although markers of insulin resistance (ΔTG/HDL-C ratio, ΔTyG index) as well as liver stiffness and steatosis indices (CAP) showed no significant change after the intervention, plasma FGF21 levels decreased despite stable BMI.

In this study, plasma FGF21 levels decreased after 12–18 months of high-protein nutritional guidance, suggesting a reduction in metabolic stress and improved anabolic efficiency.

FGF21 is secreted by both the liver and skeletal muscle, acting as an endocrine mediator of nutrient sensing and mitochondrial homeostasis.

As detailed by Li et al. [22], (Int. J. Mol. Sci., 2023; 24:16951, PMID: 38069273), FGF21 is strongly induced by mitochondrial dysfunction, endoplasmic reticulum stress, and low protein intake, and it regulates mTORC1 and AMPK pathways to restore cellular energy balance and promote muscle fiber remodeling.

Its reduction following individualized protein supplementation in our cohort likely reflects decreased catabolic signaling and improved energy status in skeletal muscle.

Together, these findings support the concept that appropriate protein intake can attenuate FGF21-mediated stress responses, thereby contributing to muscle preservation in older adults with diabetes.

Multivariate analyses further revealed that specific AAs patterns and hormonal responses, particularly those involving BCAAs, urea cycle metabolites, glucagon, and FGF21, reflect complex metabolic states that extend beyond single-marker assessments. These findings support the clinical feasibility of protein-focused dietary strategies for preserving muscle health in very elderly adults and highlight the potential value of integrated biomarker profiling in informing personalized nutritional and therapeutic approaches.

### 4.4. Clinical Implications for Adults Aged ≥ 75 Years with T2DM

The decision to focus on adults aged ≥ 75 years is clinically important, as both the prevalence and consequences of sarcopenia escalate markedly beyond this age. Epidemiological studies have shown a prevalence of approximately 22% among individuals aged 75–79 years, with sarcopenia being strongly associated with higher risks of disability and mortality [3]. In parallel, the prevalence of chronic kidney disease (CKD) rises sharply after 75 years (≈34.6% vs. 11.8% at 65–74 years) [4], making it crucial to evaluate the balance between anabolic efficacy and renal safety in this age group. Furthermore, age-related anabolic resistance, characterized by a blunted muscle protein synthesis response to dietary protein and exercise, becomes a key physiological challenge in advanced age [5]. Our findings indicate that, even among very old adults with multiple comorbidities such as diabetes and mild renal impairment, individualized nutritional guidance emphasizing adequate protein intake can stabilize or improve muscle parameters without accelerating renal decline. This supports the feasibility of high-protein counselling as a practical strategy in geriatric diabetes care, complementing physical activity interventions where possible.

In clinical practice, patients aged ≥ 75 years often present with competing priorities—maintaining independence, avoiding frailty, and preventing renal deterioration. Personalized, dietitian-led protein optimization may help balance these goals, providing a pragmatic and safe approach to sarcopenia prevention in the aging diabetic population.

This study has several strengths. It specifically targeted very old adults with T2DM, a population at particularly high risk for sarcopenia and underrepresented in prior research. Nutritional guidance was standardized and provided by dietitians, with explicit protein intake targets. Comprehensive, multi-system readouts, including muscle, renal, hepatic, and endocrine outcomes, were assessed longitudinally. Furthermore, we applied multivariate principal component analysis to characterize coordinated changes in AAs networks, providing an integrated view of metabolic adaptations.

Because this was an exploratory, integrative analysis linking nutritional, metabolic, and muscular parameters, the findings should be viewed as hypothesis-generating and will inform future focused investigations.

Nonetheless, several limitations should be acknowledged. First, the absence of a control group limits our ability to distinguish the effects of the intervention from natural age-related changes in muscle function over time. Second, because our study population consisted exclusively of adults aged ≥ 75 years with T2DM, the sample size was relatively small, which may limit the generalizability of our findings. Third, dietary intake was assessed using an FFQ, which relies on self-reported recall and may involve some degree of reporting bias, particularly in older adults. However, the FFQ was used primarily to capture recent dietary patterns and to support individualized counselling at each visit, thereby minimizing the impact of recall-related limitations. Fourth, all biochemical assessments, including plasma AAs profiles and related hormones, were conducted under fasting conditions rather than in the postprandial state. Therefore, protein intake may not have been fully reflected in circulating AAs and hormone concentrations obtained under fasting conditions, and our results may not capture acute postprandial dynamics of muscle protein synthesis. In addition, physical activity was encouraged but not systematically monitored or quantified, which may have influenced muscle-related outcomes. Although the Barthel Index was used in this study as a practical measure of general quality of life (QOL) in outpatient settings, most participants were classified as ‘almost independent’ or ‘completely independent,’ with scores ≥85. Therefore, QOL was not assessed as an intervention outcome. Nevertheless, the inclusion of more comprehensive instruments such as the SF-36, which assess both physical and mental domains of QOL, might have allowed evaluation of changes even in participants with good baseline physical function.

Future studies should include standardized assessments of physical activity and control for diabetes medication adjustments, as these lifestyle and pharmacologic factors may have affected metabolic and musculoskeletal outcomes. Moreover, because multiple statistical comparisons were performed, the possibility of type I error (false-positive findings) cannot be excluded. As the analyses were exploratory in nature, the results should be interpreted with caution and validated in future studies with larger sample sizes and pre-specified hypotheses. Furthermore, glucagon and insulin act in close physiological balance to regulate glucose and amino-acid metabolism [39]. Therefore, simultaneous evaluation of both hormones may provide a more comprehensive understanding of metabolic adaptations to high-protein nutritional guidance. Finally, future studies involving larger, multicenter cohorts and detailed assessments of postprandial AAs kinetics are required to validate our observations and to clarify the underlying mechanisms linking AAs homeostasis and sarcopenia.

## 5. Conclusions

In conclusion, long-term individualized nutritional guidance is safe and effective for mitigating sarcopenia in adults aged ≥ 75 years with T2DM. In addition, early changes in glucagon, CPR, and amino acid profiles may serve as biomarkers of the intervention response, despite their divergent relationships with muscle mass and strength that highlight the need for further studies. Our present findings provide a foundation for the development of precision nutritional strategies aimed at preserving independence and quality of life in the oldest-old with diabetes. Large-scale trials are warranted to validate and expand upon these findings and their effectiveness in maintaining independence and quality of life in this vulnerable population.

## Figures and Tables

**Table 1:** Baseline characteristics of the study participants.

Variable	All (*n* = 44)	Male (*n* = 19)	Female (*n* = 25)	*p*-Value
Age (years)	80 ± 3.8	81 ± 4.2	79 ± 3.3	0.158
Height (cm)	156.6 ± 9.5	164.3 ± 7.7	150.7 ± 5.8	<0.001 *
Weight (kg)	55.3 ± 11.4	59.2 ± 11.0	52.3 ± 11.0	0.047 *
BMI (kg/m^2^)	22.5 ± 4.2	21.8 ± 2.8	23.0 ± 5.0	0.309
Total Body Water (L)	28.9 ± 5.2	33.1 ± 4.2	25.7 ± 3.2	<0.001 *
Protein Mass (kg)	7.5 ± 1.4	8.6 ± 1.1	6.7 ± 0.8	<0.001 *
Fat Mass (kg)	16.1 ± 7.3	14.4 ± 6.6	17.4 ± 7.7	0.167
Muscle Mass (kg)	36.9 ± 6.7	42.3 ± 5.4	32.8 ± 4.1	<0.001 *
Skeletal Muscle Mass (kg)	20.7 ± 4.2	24.1 ± 3.4	18.1 ± 2.5	<0.001 *
Basal Metabolic Rate (kcal/day)	1215 ± 150.6	1337 ± 123.8	1122 ± 92.2	<0.001 *
SMI (kg/m^2^)	6.2 ± 1.1	6.8 ± 0.8	5.8 ± 1.0	<0.001 *
Grip Strength (kg)	23.7 ± 6.7	29.3 ± 5.4	19.5 ± 3.8	<0.001 *
Gait Speed (m/s)	0.88 ± 0.20	0.87 ± 0.19	0.88 ± 0.20	0.896
Barthel Index (score)	95 ± 8	97 ± 5	94 ± 9	0.157
Systolic BP (mmHg)	131 ± 19.1	123 ± 12.3	138 ± 20.8	0.004 *
Diastolic BP (mmHg)	72 ± 11.3	72 ± 8.8	73 ± 13.1	0.816
AST (U/L)	23 ± 9.3	23 ± 6.3	23 ± 11.2	0.954
ALT (U/L)	19 ± 11.7	20 ± 10.7	19 ± 12.5	0.705
GGT (U/L)	22 ± 13.7	22 ± 14.3	22 ± 13.5	0.986
FIB4 Index	2.37 ± 0.79	2.43 ± 0.67	2.33 ± 0.89	0.660
Liver Stiffness (kPa)	4.3 ± 1.6	4.5 ± 2.0	4.2 ± 1.2	0.602
CAP (dB/m)	206.5 ± 48.2	205.0 ± 62.9	207.6 ± 35.8	0.878
Urea Nitrogen (mg/dL)	17.4 ± 6.3	18.9 ± 6.2	16.3 ± 6.3	0.188
Creatinine (mg/dL)	0.75 ± 0.24	0.88 ± 0.23	0.65 ± 0.19	<0.001 *
eGFRcreat (mL/min/1.73 m^2^)	69.7 ± 19.4	66.6 ± 15.1	72.1 ± 22.1	0.337
Cystatin C(mg/L)	1.07 ± 0.27	1.10 ± 0.26	1.04 ± 0.28	0.453
eGFRcys (mL/min/1.73 m^2^)	64.1 ± 18.4	63.4 ± 16.1	64.6 ± 20.3	0.823
Uric Acid (mg/dL)	4.8 ± 1.2	5.3 ± 1.1	4.3 ± 1.1	0.003 *
Plasma Glucose (mg/dL)	141 ± 34.6	134 ± 39.6	145 ± 30.3	0.306
HbA1c (NGSP) (%)	7.9 ± 1.5	7.7 ± 1.2	8.1 ± 1.7	0.466
CPR (ng/mL)	1.43 ± 0.93	1.54 ± 0.99	1.34 ± 0.90	0.512
Glucagon (pg/mL)	19.4 ± 10.6	21.9 ± 11.6	17.6 ± 9.6	0.201
FGF21 (pg/mL)	223.2 ± 168.9	219.5 ± 189.6	226.1 ± 155.4	0.904
Triglycerides (mg/dL)	92 ± 35.0	92 ± 43.7	92 ± 27.7	0.964
Total Cholesterol (mg/dL)	174 ± 34.3	166 ± 33.0	180 ± 34.5	0.171
HDL-C (mg/dL)	49 ± 16.4	44 ± 10.1	53 ± 19.3	0.056
LDL-C (mg/dL)	99 ± 26.1	97 ± 30.6	100 ± 22.6	0.657
FFA (μEq/L)	718.4 ± 191.3	685.0 ± 229.3	743.8 ± 156.8	0.344
Total Protein (g/dL)	6.9 ± 0.5	7.0 ± 0.4	6.79 ± 0.49	0.143

Data are presented as mean ± standard deviation (SD). *p*-values were calculated using independent *t*-tests comparing male and female participants. Values with *p* < 0.05 were considered statistically significant and are marked with an asterisk (*). Units are expressed in conventional or SI units according to standard clinical reporting practices. Abbreviations: BMI, body mass index; BP, blood pressure.

**Table 2:** Comparison of baseline nutrient intake and recommended nutrient targets.

Nutrient Type	Baseline Intake (Mean ± SD)	Recommended Intake (Mean ± SD)	*p*-Value
Protein (g/kgIBW)	1.26 ± 0.37	1.41 ± 0.23	0.028 *
Energy (kcal)	1680 ± 350	1640 ± 160	0.460
Calcium (mg)	553 ± 206	643 ± 50	0.008 *
Vitamin D (μg)	7.7 ± 4.0	8.5 ± 0.0	0.200

All nutrient values represent daily intake. Abbreviations: IBW, ideal body weight. Note: Values are mean ± SD. * p < 0.05 by paired *t*-test (vs. target). Protein, calcium, and vitamin D targets were defined by individualized dietary guidance as described in Methods.

**Table 4:** Correlations between CPR, Glucagon, FGF21 and Clinical Variables.

Variable	Univariate Analysis				Multivariate Analysis	
	Non-Adjusted		Adjusted For Age And Sex			
	r	P (r)	Partial r	P (Partial r)	β (95%CI)	P (β)
(a) C-peptide (CPR)-related variables						
BMI	0.59	<0.001 ***	0.62	<0.001 ***	0.62 (0.35–0.87)	<0.001 ***
Fat Mass (kg)	0.71	<0.001 ***	0.74	<0.001 ***	0.73 (0.51–0.95)	<0.001 ***
Body fat percentage (%BF)	0.55	<0.001 ***	0.64	<0.001 ***	0.59 (0.35–0.82)	<0.001 ***
SMI (kg/m^2^)	0.52	<0.001 ***	0.53	<0.001 ***	0.47 (0.21–0.71)	<0.001 ***
Grip Strength (kg)	0.12	0.444	0.04	0.817	0.02 (−0.19–0.24)	0.821
Gait Speed (m/s)	−0.12	0.468	−0.15	0.36	−0.14 (−0.47–0.18)	0.373
AST (U/L)	0.26	0.096	0.25	0.106	0.26 (−0.06–0.57)	0.116
ALT (U/L)	0.35	0.023 *	0.33	0.03 *	0.34 (0.02–0.64)	0.035 *
GGT (U/L)	0.44	0.004 **	0.47	0.002 **	0.47 (0.18–0.76)	0.002 **
FIB4 Index	−0.04	0.81	−0.01	0.943	−0.01 (−0.33–0.31)	0.945
Urea Nitrogen (mg/dL)	0.24	0.128	0.25	0.106	0.25 (−0.06–0.56)	0.115
Creatinine (mg/dL)	0.53	<0.001 ***	0.58	<0.001 ***	0.51 (0.27–0.75)	<0.001 ***
eGFRcreat (mL/min/1.73 m^2^)	−0.5	0.001 **	−0.54	<0.001 ***	−0.53 (−0.81–−0.25)	<0.001 ***
Cystatin C (mg/L)	0.37	0.017 *	0.41	0.007 **	0.41 (0.11–0.69)	0.008 **
eGFRcys (mL/min/1.73 m^2^)	−0.34	0.029 *	−0.42	0.006 **	−0.40 (−0.68–−0.11)	0.007 **
Uric Acid (mg/dL)	0.27	0.09	0.28	0.078	0.25 (−0.03–0.53)	0.086
Plasma Glucose (mg/dL)	0.26	0.097	0.27	0.088	0.27 (−0.049–0.58)	0.096
HbA1c (NGSP) (%)	−0.09	0.554	−0.09	0.557	−0.09 (−0.41–0.23)	0.567
Glucagon (pg/mL)	0.14	0.366	0.15	0.352	0.15 (−0.17–0.46)	0.364
FGF21 (pg/mL)	0.34	0.029 *	0.34	0.028 *	0.34 (0.03–0.65)	0.032 *
Triglycerides (mg/dL)	0.54	<0.001 ***	0.55	<0.001 ***	0.56 (0.28–0.83)	<0.001 ***
Total Cholesterol (mg/dL)	−0.25	0.11	−0.23	0.146	−0.23 (−0.54–0.09)	0.156
HDL-C (mg/dL)	−0.4	0.008 **	−0.39	0.012 *	−0.38 (−0.6–−0.08)	0.014 *
LDL-C (mg/dL)	−0.16	0.307	−0.16	0.324	−0.16 (−0.48–0.17)	0.336
FFA (μEq/L)	0.02	0.92	0.01	0.946	0.01 (−0.31–0.3)	0.948
(b) Glucagon-related variables						
BMI	0.12	0.438	0.17	0.286	0.17 (−0.15–0.48)	0.298
Fat Mass (kg)	0.21	0.182	0.28	0.071	0.28 (−0.03–0.58)	0.078
Body fat percentage (%BF)	0.16	0.301	0.29	0.061	0.27 (−0.02–0.55)	0.068
SMI (kg/m^2^)	0.17	0.284	0.1	0.517	0.09 (−0.19–0.37)	0.528
Grip Strength (kg)	0.27	0.077	0.22	0.149	0.15 (−0.06–0.35)	0.16
Gait Speed (m/s)	0.08	0.613	0.09	0.562	0.09 (−0.24–0.42)	0.573
AST (U/L)	−0.02	0.886	−0.02	0.881	−0.02 (−0.35–0.30)	0.884
ALT (U/L)	−0.05	0.771	−0.05	0.754	−0.05 (−0.37–0.27)	0.76
GGT (U/L)	−0.12	0.438	−0.13	0.398	−0.13 (−0.46–0.19)	0.41
FIB4 Index	0.07	0.659	0.03	0.835	0.03 (−0.28–0.35)	0.839
Urea Nitrogen (mg/dL)	0.3	0.052	0.26	0.088	0.26 (−0.04–0.57)	0.096
Creatinine (mg/dL)	0.28	0.073	0.21	0.177	0.19 (−0.09–0.47)	0.188
eGFRcreat (mL/min/1.73 m^2^)	−0.19	0.222	−0.16	0.306	−0.16 (−0.47–0.15)	0.318
Cystatin C (mg/L)	0.16	0.3	0.13	0.405	0.13 (−0.18–0.44)	0.417
eGFRcys (mL/min/1.73 m^2^)	−0.08	0.615	−0.05	0.747	−0.05 (−0.36–0.26)	0.753
Uric Acid (mg/dL)	0.04	0.808	−0.06	0.694	−0.06 (−0.35–0.23)	0.701
Plasma Glucose (mg/dL)	−0.06	0.691	−0.01	0.924	−0.01 (−0.33–0.30)	0.926
HbA1c (NGSP) (%)	−0.21	0.168	−0.19	0.213	−0.20 (−0.51–0.12)	0.224
CPR (ng/mL)	0.14	0.366	0.15	0.352	0.15 (−0.17–0.47)	0.364
FGF21 (pg/mL)	0.06	0.722	0.07	0.649	0.07 (−0.25–0.40)	0.657
Triglycerides (mg/dL)	0.12	0.459	0.13	0.411	0.13 (−0.19–0.45)	0.423
Total Cholesterol (mg/dL)	−0.01	0.96	0.02	0.887	0.02 (−0.30–0.34)	0.89
HDL-C (mg/dL)	−0.06	0.685	−0.03	0.835	−0.03 (−0.35–0.28)	0.839
LDL-C (mg/dL)	0.01	0.931	0.03	0.853	0.03 (−0.30–0.36)	0.856
FFA (μEq/L)	0.18	0.253	0.24	0.116	0.24 (−0.07–0.55)	0.125
(c) Fibroblast growth factor 21 (FGF21)-related variables						
BMI	0.15	0.322	0.15	0.347	0.14 (−0.17–0.46)	0.359
Fat Mass (kg)	0.31	0.041 *	0.31	0.045 *	0.30 (−0.00–0.59)	0.051
Body fat percentage (%BF)	0.23	0.134	0.23	0.135	0.21 (−0.07–0.49)	0.145
SMI (kg/m^2^)	0.03	0.862	0.04	0.782	0.04 (−0.24–0.32)	0.788
Grip Strength (kg)	−0.21	0.168	−0.3	0.05	−0.20 (−0.39–0.00)	0.056
Gait Speed (m/s)	−0.4	0.009 **	−0.42	0.006 **	−0.42 (−0.71–−0.11)	0.008 **
AST (U/L)	0.11	0.486	0.11	0.497	0.11 (−0.21–0.42)	0.508
ALT (U/L)	0.06	0.716	0.05	0.738	0.05 (−0.26–0.37)	0.744
GGT (U/L)	0.21	0.173	0.22	0.155	0.22 (−0.09–0.53)	0.166
FIB4 Index	−0.14	0.368	−0.13	0.424	−0.12 (−0.43–0.18)	0.435
Urea Nitrogen (mg/dL)	−0.01	0.946	0.0	0.977	0.00 (−0.31–0.32)	0.977
Creatinine (mg/dL)	0.18	0.235	0.24	0.124	0.21 (−0.06–0.48)	0.134
eGFRcreat (mL/min/1.73 m^2^)	−0.1	0.507	−0.12	0.428	−0.12 (−0.43–0.19)	0.44
Cystatin C (mg/L)	0.37	0.015 *	0.4	0.008 **	0.39 (0.10–0.67)	0.009 **
eGFRcys (mL/min/1.73 m^2^)	−0.37	0.013 *	−0.42	0.005 **	−0.40 (−0.67–−0.12)	0.006 **
Uric Acid (mg/dL)	0.04	0.814	0.07	0.668	0.06 (−0.22–0.35)	0.676
Plasma Glucose (mg/dL)	−0.08	0.613	−0.1	0.524	−0.10 (−0.41–0.21)	0.534
HbA1c (NGSP) (%)	−0.25	0.102	−0.26	0.09	−0.26 (−0.57–0.05)	0.099
Glucagon (pg/mL)	0.06	0.722	0.07	0.649	0.07 (−0.24–0.38)	0.657
CPR (ng/mL)	0.34	0.029	0.34	0.028 *	0.33 (0.02–0.63)	0.032 *
Triglycerides (mg/dL)	0.4	0.009 **	0.39	0.009 **	0.39 (0.09–0.69)	0.011 *
Total Cholesterol (mg/dL)	0.19	0.231	0.19	0.227	0.19 (−0.12–0.49)	0.239
HDL-C (mg/dL)	−0.05	0.773	−0.05	0.767	−0.04 (−0.35–0.26)	0.772
LDL-C (mg/dL)	0.18	0.251	0.18	0.258	0.18 (−0.14–0.49)	0.27
FFA (μEq/L)	0.19	0.226	0.17	0.262	0.17 (−0.13–0.47)	0.274

Pearson’s correlation coefficients (r), partial correlation coefficients adjusted for age and sex (partial r), and standardized regression coefficients (β) with 95% confidence intervals (CI) were calculated for each variable. Sex was coded as 0 (male) and 1 (female). * p < 0.05, ** p < 0.01, *** p < 0.001. Abbreviations: AST, aspartate aminotransferase; ALT, alanine aminotransferase; BMI, body mass index; eGFRcreat, estimated glomerular filtration rate based on creatinine; eGFRcys, eGFR based on cystatin C; FFA, free fatty acid; FIB4, fibrosis-4 index; GGT gamma-glutamyl transferase; HDL-C, high density lipoprotein cholesterol; LDL-C, low density lipoprotein cholesterol; SMI, skeletal mass index.

## Data Availability

The original contributions presented in the study are included in the article, further inquiries can be directed to the corresponding author.

## Supplementary Materials

The following supporting information can be downloaded at: https://www.mdpi.com/article/10.3390/nu17213459/s1, Table S1: Full multiple linear regression models for changes in sarcopenia-related indices.; Table S2: Changes in the consumption of protein-rich food groups assessed by a food frequency questionnaire (FFQg). Table S3: Scheme S3. (a–c). Associations between Sarcopenia-Related Parameters with circulating branched-chain amino acid (BCAA) concentrations at each study visit, stratified by sex.
