# Supplementary material for: Effects of High-Protein Nutritional Guidance on Sarcopenia-Related Parameters in Individuals Aged ≥ 75 Years with Type 2 Diabetes: An Exploratory Single-Arm Pre–Post Intervention Study
Source: Nutrients. 2025 Nov 1;17(21):3459. doi: 10.3390/nu17213459 (PMC12608599; doi:10.3390/nu17213459)
Supplement: Supplementary file 1 [file nutrients-17-03459-s001.zip › nutrients-3935132-supplementary.pdf]

Supplementary

Table S1: Full multiple linear regression models for changes in sarcopenia-related indices

Dependent variable	Independent variable	β	SE	Std. β	t	p-value
**ΔnSMI**	Sex	1.91	1.59	0.18	1.20	0.239
	Age	−0.07	0.21	−0.05	−0.32	0.752
	ΔGlucagon	0.16	0.07	0.41	2.45	0.020*
	ΔCPR	−2.81	1.16	−0.41	−2.42	0.022*
	PC8	−2.46	0.80	−0.42	−3.07	0.005
	PC2	−0.38	0.86	−0.06	−0.44	0.666
	PC3	0.66	0.53	0.19	1.24	0.226
	PC9	−0.98	0.85	−0.16	−1.15	0.259
**ΔnGrip**	Sex	−4.79	4.73	−0.15	−1.01	0.320
	Age	0.21	0.61	0.05	0.34	0.739
	ΔGlucagon	−0.40	0.43	−0.09	−0.94	0.028*
	ΔCPR	−0.78	1.35	−0.04	−0.58	0.832
	PC1	−2.09	0.80	−0.36	−2.64	0.013*
	PC7	−5.28	2.25	−0.39	−2.34	0.026*
	PC3	−3.82	2.05	−0.42	−1.87	0.074
	PC2	−2.05	1.48	−0.23	−1.39	0.174

Model fit (ΔnSMI): R = 0.668, R^2 = 0.447, Adj.R^2 = 0.299, F(8,30) = 3.025, p = 0.013
Model fit (ΔnGrip): R = 0.683, R^2 = 0.466, Adj.R^2 = 0.324, F(8,30) = 3.273, p = 0.008
Abbreviations: CPR, C-peptide; PC, principal component; SE, standard error; Std. β, standardized regression coefficient.
Note: *p < 0.05, **p < 0.01.

Table S2. Changes in the consumption of protein-rich food groups assessed by a food frequency questionnaire (FFQg) at baseline (V1), 6 months (V2), 12 months (V3), and 18 months (V4).

Food group	V1 (g/day)	V2 (g/day)	V3 (g/day)	V4 (g/day)
Legumes	66.3 ± 45.9	65.2 ± 47.9	66.0 ± 47.3	69.6 ± 46.8
Fish and seafood	78.6 ± 50.0	72.1 ± 48.8	70.9 ± 50.2	69.1 ± 44.2
Meat and meat products	63.8 ± 58.8	55.6 ± 42.0	60.1 ± 50.4	59.8 ± 44.9
Eggs	46.6 ± 31.0	42.0 ± 30.7	45.5 ± 32.9	46.8 ± 34.4
Dairy products	168.0 ± 100.1	191.5 ± 96.6*	170.9 ± 105.4	172.3 ± 142.1

Data are presented as mean ± standard deviation (SD), in grams per day (g/day). Paired t-tests were performed to compare each follow-up visit (V2–V4) with baseline (V1). *Significant difference from baseline (p = 0.007).

Supplementary Table S3.
Table S3(a). Associations between skeletal muscle mass index (SMI) and circulating BCAA concentrations at each study visit, stratified by sex.

Dep var.	Ind var.	All r (p)	Adj r (p)	All β (p)	M r (p)	M β (p)	F r (p)	F β (p)
SMI _V1	BCAA _V1	**0.35 (0.02)**	0.13 (0.39)	0.00 (0.40)	0.23 (0.35)	0.00 (0.28)	0.14 (0.52)	-0.01 (0.19)
SMI _V2	BCAA _V2	0.23 (0.15)	0.11 (0.49)	0.00 (0.51)	0.07 (0.79)	0.00 (0.69)	0.17 (0.45)	-0.00 (0.77)

SMI _V3	BCAA _V3	**0.42 (0.01)**	**0.33 (0.03)**	**0.00 (0.04)**	0.13 (0.60)	0.00 (0.77)	**0.45 (0.03)**	0.00 (0.86)
SMI _V4	BCAA _V4	0.20 (0.23)	-0.12 (0.46)	-0.00 (0.47)	0.01 (0.97)	0.00 (0.49)	-0.13 (0.58)	-0.01 (0.15)

Plasma BCAA concentrations were positively correlated with skeletal muscle mass index (SMI), most notably at 12 months (V3). After adjustment for age and sex, the association remained significant (partial r = 0.33, p = 0.03; β = 0.00 [95% CI 0.00–0.01], p = 0.04). No significant relationships were observed at other time points. These findings suggest that circulating BCAA levels may contribute to muscle mass maintenance in older adults with type 2 diabetes.

Table S3(b). Associations between grip strength and circulating BCAA concentrations at each study visit, stratified by sex.

**Dep var.**	**Ind var.**	**All r (p)**	**Adj r (p)**	**All β (p)**	**M r (p)**	**M β (p)**	**F r (p)**	**F β (p)**
Grip strength _V1	BCAA _V1	**0.39 (0.01)**	0.02 (0.89)	0.00 (0.89)	0.24 (0.32)	0.01 (0.52)	-0.11 (0.61)	0.01 (0.67)
Grip strength _V2	BCAA _V2	0.18 (0.28)	-0.13 (0.42)	-0.01 (0.44)	0.01 (0.97)	-0.03 (0.24)	-0.11 (0.62)	0.01 (0.62)
Grip strength _V3	BCAA _V3	0.27 (0.08)	0.02 (0.91)	0.00 (0.91)	0.07 (0.77)	0.01 (0.55)	0.15 (0.48)	0.01 (0.48)
Grip strength _V4	BCAA _V4	**0.36 (0.03)**	-0.10 (0.56)	-0.01 (0.57)	-0.02 (0.95)	-0.01 (0.56)	-0.00 (0.99)	0.01 (0.57)

Across all study visits, **significant positive associations were observed at baseline (V1) and at 18 months (V4)**, whereas no significant relationships were found at 6 or 12 months. However, after adjustment for age and sex, these associations became non-significant. Similarly, in sex-stratified analyses, no significant relationships were identified. These results indicate that **while higher plasma BCAA concentrations may be linked to greater grip strength in unadjusted analyses**, this relationship appears to be largely explained by age and sex.

Table S3(c). Associations between gait speed and circulating BCAA concentrations at each study visit, stratified by sex.

**Dep var.**	**Ind var.**	**All r (p)**	**Adj r (p)**	**All β (p)**	**M r (p)**	**M β (p)**	**F r (p)**	**F β (p)**
Gait speed _V1	BCAA _V1	-0.28 (0.08)	**-0.36 (0.02)**	**-0.00 (0.02)**	-0.15 (0.55)	-0.00 (0.08)	**-0.44 (0.04)**	0.00 (0.97)
Gait speed _V2	BCAA _V2	0.00 (0.98)	-0.10 (0.54)	-0.00 (0.56)	0.06 (0.83)	-0.00 (0.47)	-0.03 (0.90)	0.00 (0.31)
Gait speed _V3	BCAA _V3	-0.13 (0.42)	**-0.39 (0.01)**	**-0.00 (0.02)**	-0.09 (0.73)	-0.00 (0.09)	-0.40 (0.07)	-0.00 (0.46)
Gait speed _V4	BCAA _V4	0.06 (0.73)	-0.01 (0.96)	-0.00 (0.96)	0.30 (0.24)	-0.00 (0.99)	-0.10 (0.69)	0.00 (0.21)

Negative associations were observed between BCAA concentrations and gait speed at baseline (V1) and 12 months (V3) after adjustment for age and sex (V1: partial r = −0.37, p = 0.02; β = −0.00 [95% CI −0.00 to −0.00], p = 0.02; V3: partial r = −0.39, p = 0.01; β = −0.00 [95% CI −0.00 to −0.00], p = 0.02). Among women, this inverse relationship was more pronounced (r = −0.44, p = 0.04 at baseline), while no consistent associations were found in men. These findings suggest that higher circulating BCAA levels may be linked to slower gait speed, particularly among older women.

Legend (Tables S3 a–c).

Simple and partial correlation analyses, as well as multivariable regression models, were performed to assess the relationships between muscle-related parameters (SMI, grip strength, and gait speed) and plasma branched-chain amino acid (BCAA) concentrations at baseline (V1), 6 months (V2), 12 months (V3), and 18 months (V4). Partial correlations and regression models were adjusted for age and sex in the overall analyses, and for age only in the sex-stratified analyses. Values are presented as correlation coefficients (r) with corresponding p-values and regression coefficients (β) with 95% confidence intervals (CI) and p-values. Bold values indicate statistically significant associations ($p < 0.05$).

Interpretation: Circulating BCAA concentrations were positively associated with skeletal muscle mass and showed modest relationships with muscle strength and physical performance. The inverse associations observed between BCAA levels and gait speed should not be interpreted as impaired function but rather as a reflection of physiological mechanics—greater muscle mass entails larger body mass and inertia, which may modestly reduce movement velocity. Taken together, these findings suggest that BCAAs contribute to both muscle quantity and functional capacity, serving as a metabolic indicator of overall muscle health in older adults with type 2 diabetes.
